# A Comparison of the Efficacy of Voltage-directed Cavotricuspid Isthmus Ablation Using Mini Versus Conventional Electrodes

**DOI:** 10.19102/icrm.2018.090603

**Published:** 2018-06-15

**Authors:** Riyaz Somani, G. Andre Ng, Niel A. Hobson, Damian P. Redfearn, Jane C. Caldwell

**Affiliations:** ^1^Department of Cardiology, Glenfield Hospital, Leicester, UK; ^2^NIHR Biomedical Research Centre, University of Leicester, Leicester, UK; ^3^Department of Cardiology, Castle Hill Hospital, Hull and East Yorkshire NHS Trust, Kingston-upon-Hull, UK; ^4^Department of Medicine, Queen’s University, Kingston, Ontario, Canada; ^5^Hull York Medical School, University of Hull, Kingston-upon-Hull, UK

**Keywords:** Atrial flutter, catheter ablation, voltage-directed technique

## Abstract

Cavotricuspid isthmus (CTI) ablation is a current first-line management method for typical atrial flutter. A voltage-directed technique that systematically targets points of maximal voltage has be found to reduce procedure and fluoroscopy times without increasing recurrence. We hypothesized that this technique’s efficiency would be enhanced by using signals from radial minielectrodes of a novel catheter (IntellaTip MiFi™; Boston Scientific, Natick, MA, USA). Prospectively, atrial flutter patients underwent voltage-directed ablation with a nonirrigated 8-mm-tip catheter. Ablation was either directed by conventional bipolar electrodes (group A, n = 13) or mini-electrodes (group B, n = 17) with the goal of achieving bidirectional block at the CTI and a subsequent observation time of 30 minutes. Total radiofrequency application time and lesion numbers were not significantly different. Group B had a lower mean power [38.7 watts (W) ± 2.0 W versus 44.8 W ± 1.9 W; p < 0.05] and a tendency for longer fluoroscopy and procedure times. In three of the cases in group B, a switch to an irrigated catheter was required in order to achieve bidirectional block. In group A, bidirectional block was obtained in all patients using the nonirrigated catheter with no significant increase in reconnection. Differences in the catheter performance between the two groups were driven by poorer performance of the MiFi™ catheter (Boston Scientific, Natick, MA, USA) in patients presenting in atrial flutter. Electroanatomical mapping revealed a more proximal localization of the maximal voltage by the minielectrodes as compared with the conventional bipolar electrodes, resulting in less efficient identification and ablation of the conducting muscle bundles. Final results indicated CTI ablation using minielectrodes is not superior to conventional bipolar electrodes in the use of 8-mm, nonirrigated electrodes.

## Introduction

Cavotricuspid isthmus (CTI) ablation is now a first-line management technique for CTI-dependent atrial flutter, a condition with an incidence of 88 per 100,000 patients-years in the general population. Radiofrequency ablation by a voltage-direction strategy, which systematically targets points of maximal voltage across the CTI, has been shown to reduce procedure and fluoroscopy times without increasing recurrence rates.^1–3^

**Figure 1:**
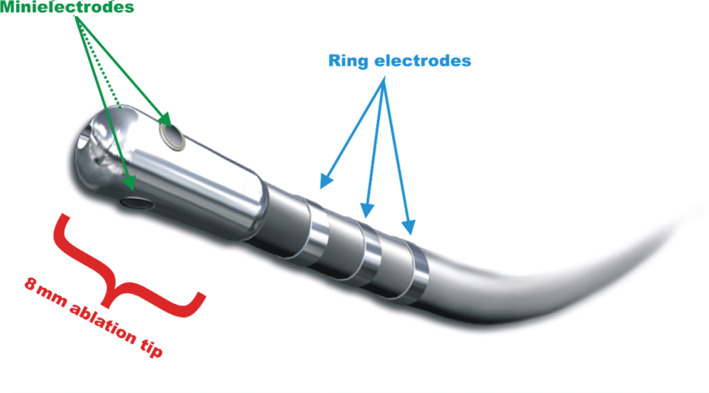
Annotated picture of the IntellaTip MiFi™ catheter (Boston Scientific, Natick, MA, USA).

A novel ablation catheter, the IntellaTip MiFi™ (Boston Scientific, Natick, MA, USA; **Figure 1**), with three mini electrodes measuring 0.8 mm in diameter that are arranged radially 1.3 mm from the tip, has been shown in animal studies to demonstrate enhanced detection of electrical signals^4^ and, in humans, to improve the detection of viable tissue in redo flutter procedures^5^ and in the ablation of atriofascicular pathways, respectively.^6^

We thus hypothesized that using the minielectrodes in this catheter would further enhance the voltage-directed technique during CTI ablation.

## Methods

In two large, tertiary centers, data were collected prospectively in consecutive atrial flutter patients undergoing ablation. The patients underwent voltage-directed CTI ablation using an 8-mm-tip, nonirrigated ablation catheters without the assistance of electroanatomical mapping as per the standard technique. This technique has been previously described^1–3^ but, briefly, the operator maps across the CTI for the point of highest voltage for targeting ablation sequentially until bidirectional block is achieved. This study was a nonrandomized, service evaluation of new equipment using a standard technique. This study was discussed with the local ethical committee, who deemed that ethical approval was not required given the use of an accepted technique as standard with the service evaluation of a new catheter. Upon receipt of the MiFi™ catheters (Boston Scientific, Natick, MA, USA), all subsequent typical atrial flutter ablations were performed with their involvement until all of the provided supplies were utilized. The subsequent typical flutter ablations were performed using an identical catheter without the minielectrodes, ie, the 8-mm Prime Blazer™ (Boston Scientific, Natick, MA, USA). Data in all ablations were collected prospectively. The cohorts were separated by catheter used. In group A (n = 13; with six patients in flutter at the time of the procedure), ablation was guided by the conventional electrodes in the Prime Blazer™ catheter (Boston Scientific, Natick, MA, USA). In group B (n = 17; with eight patients in flutter at the time of the procedure), ablation was guided by the signals from the minielectrodes located 2 mm from the tip of the catheter in the IntellaTip MiFi™ catheter (Boston Scientific, Natick, MA, USA), whose platform is identical to that of the Prime Blazer™ catheter (Boston Scientific, Natick, MA, USA) used in group A. In both groups, ablation was temperature-limited with an initial power of 60 watts (W) and a temperature of 60°C. Three experienced operators from two separate centers carried out the ablation procedures in both groups.

Patient demographics were collected from medical records and ablation, fluoroscopy, and procedure details were collated locally in each department. Data from all procedures across the two centers were then combined and statistical analysis was performed by unpaired t-test within Excel (Microsoft Corp., Redmond, WA, USA) and Fisher’s exact test. Data are presented in the format of mean ± standard error.

## Results

As detailed in **Table 1**, total radiofrequency (RF) ablation time and number of applications were not significantly different between the two groups. The MiFi™ catheter (Boston Scientific, Natick, MA, USA) was associated with significantly lower powers achieved (38.7 W ± 2.0 W versus 44.8 W ± 1.9 W; p < 0.05) and a tendency towards longer fluoroscopy (21.5 minutes ± 3.0 minutes versus 15.8 minutes ± 2.0 minutes) and procedure times (100.6 minutes ± 11.8 minutes versus 82.0 minutes ± 4.8 minutes) in group B. In three cases, bidirectional block was not obtained with the MiFi™ catheter (Boston Scientific, Natick, MA, USA) and a switch to a 4-mm irrigated tip (FlexAbility™; Abbott Laboratories, Chicago, IL, USA) had to be made in order to achieve this. No such switches were required with use of the conventional 8-mm catheter in group A.

An analysis of outcomes according to presenting rhythm appears to show that the differences in ablation were driven by poorer performance of the MiFi™ catheter (Boston Scientific, Natick, MA, USA) in patients presenting in atrial flutter. As shown in **Table 2**, patients who were in flutter at the start of the procedure had statistically lower powers in group B as compared with in group A (37.1 W ± 2.3 W versus 46.0 W ± 2.9 W; p < 0.05), as well as longer fluoroscopy times (26.3 minutes ± 4.8 minutes versus 13.4 minutes ± 2.7 minutes; p < 0.05) and longer procedure times (115.7 minutes ± 17.7 minutes versus 73 minutes ± 6.9 minutes; p < 0.05). Those patients in flutter also showed significantly higher temperatures in group B as compared with in group A (55.2°C ± 1.1°C versus 51.8°C ± 0.6°C; p < 0.05). By comparison, there were no statistical differences in any of the parameters between the groups for patients presenting in sinus rhythm **(Table 3)**.

**Figure 2:**
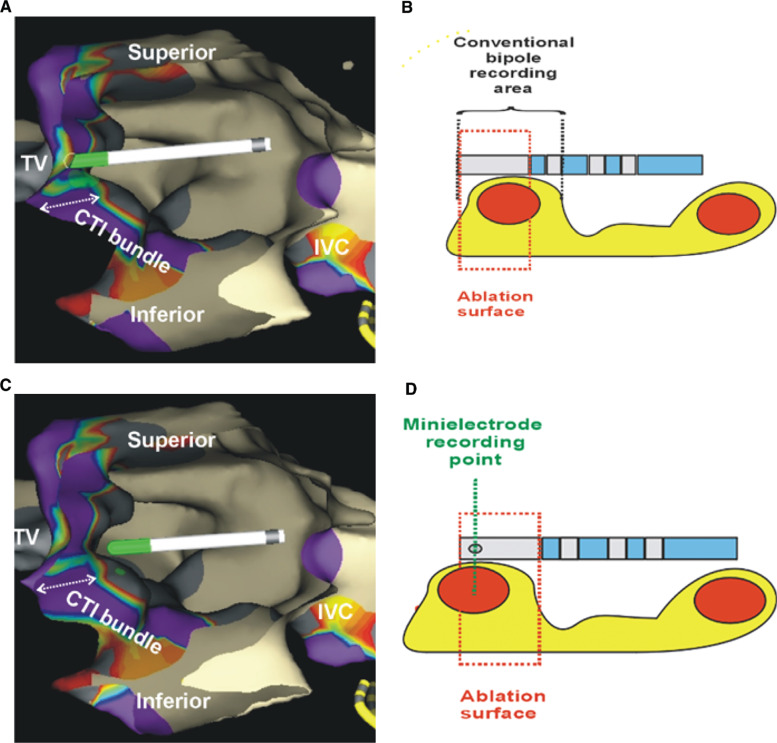
Mapping of voltage across the CTI during pacing from a coronary sinus catheter using the EnSite™ Velocity™ system (Abbott Laboratories, Chicago, IL, USA). **A**: The catheter location at maximal point with conventional bipolar mapping. **B:** A diagrammatic representation of the catheter in **A** with respect to the underlying muscle bundle. **C:** The catheter’s location at maximal point with minielectrode signals. **D:** A diagrammatic representation of the catheter in **C** with respect to the underlying muscle bundle.

Electroanatomical mapping (EAM) (EnSite™ Velocity™; Abbott Laboratories, Chicago, IL, USA) was performed in a single case to compare the location of the catheter on mapping signals according to conventional electrodes **(Figures 2A and 2B)** versus the minielectrodes **(Figures 2C and 2D)**. The first panel in **Figure 2** shows a cut-away view of the ablation catheter on the CTI during pullback with the catheter at the point of maximal voltage signal. This showed a more caval location of the catheter on mapping with minielectrodes.

## Discussion

Catheter ablation of the CTI is a well-established and curative first-line therapy for patients with typical atrial flutter with long-term success rates of ~90%.^7^ Recurrence of CTI-dependent flutters postablation are due to reconduction through the CTI.^8^ One potential mechanism for this conduction recovery is insufficient effective ablation of the conducting fibers and with lack of appreciation of this due to attendant tissue edema associated with ablation. In an attempt to avoid unnecessary edema, a voltage-dependent technique was developed and verified in London, Ontario, Canada.^1–3^

**Figure 3:**
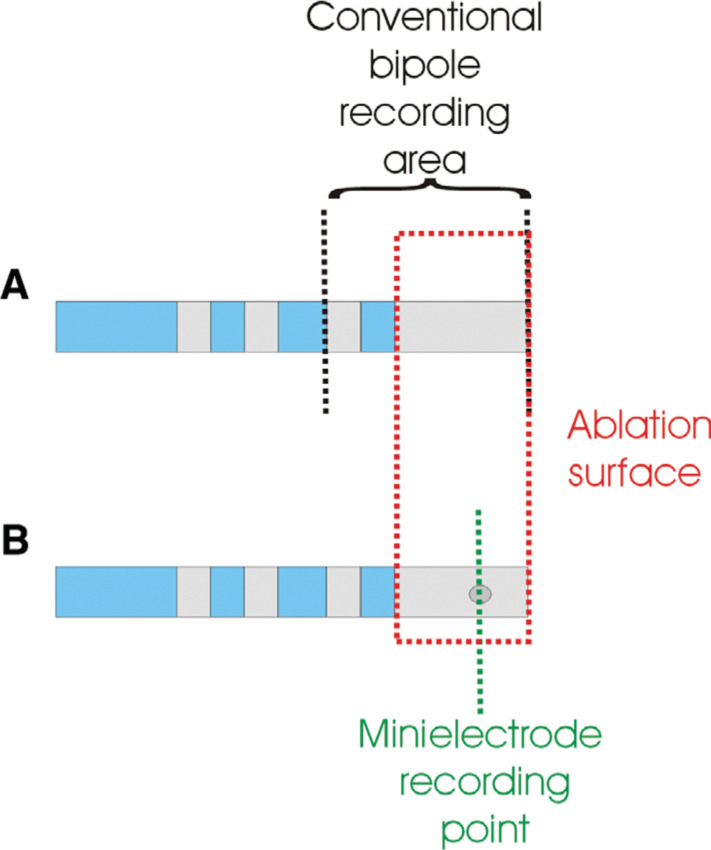
A diagrammatic representation of the recording area of a conventional bipole (top) versus the minielectrode (bottom).

The concept of the voltage-directed technique is to target conducting bundles of the CTI whilst avoiding intervening nonconducting fibrous tissue.^1^ With conventional bipolar 8-mm-tip ablation catheters, the highest voltage from the distal bipole is measured over a comparatively large surface area, the midpoint of which is towards the proximal end of the ablation area **(Figure 3)**. Theoretically, this will result in the large portion of the ablation lesion being applied slightly ahead of the highest signal. By comparison, the MiFi™ recording electrode is situated at the distal tip of the 8-mm electrode **(Figure 3)**.

In this study, we report a lack of benefit with the use of minielectrodes in voltage-directed CTI ablation and thereby provide some insights into the lack of efficacy, in keeping with other research that found no benefit of the IntellaTip MiFi™ catheter (Boston Scientific, Natick, MA, USA) in linear drag lesion technique ablation of atrial flutter.^9^ We found that careful mapping of voltages in the absence of an EAM was surprisingly associated with longer procedural times and, in three occasions, a switch to an irrigated catheter was necessary. Similarly, Iwasawa et al. found that the use of the minielectrode catheter was associated with the need for a significantly higher number of RF energy applications and longer fluoroscopic and procedure times as compared with results in cohorts in which an 8-mm dumbbell catheter or 8-mm cryocatheter were used.^9^ In both linear drag and targeted CTI ablation, the operator is guided by local bipolar signals, positioning the catheter at the optimal candidate signal with the assumption that this is also the point of maximal ablation delivery. This is highly dependent on the catheter orientation and design, positioning of recording electrodes, and the electrodes’ relationship with the ablation tip. In this study, we show a lack of benefit of use of an 8-mm-tip in CTI ablation; the explanation for this is illustrated by the EAM use shown in **Figure 2**. Mapping with minielectrodes resulted in a more caval location of the catheter tip, so the RF energy would be delivered somewhat proximal to the optimal signal, whereas, in a conventional bipole configuration, the ablation is slightly distal in a typical catheter orientation on the CTI, wherein the electrodes are parallel rather than perpendicular to the tissue. Such a location mismatch, particularly in a targeted, voltage-guided procedure, would deliver less-sustained tissue contact, as the catheter would intermittently be opposed and unopposed on the “muscle bundle” **(Figures 2C and 2D)**, whereas, by comparison, the conventional bipole mapping would result in a catheter that rocks across the “muscle bundle” with cardiac motion **(Figures 2A and 2B)**. This mechanism is supported by the lower powers and lower efficiency of the ablation strategy in cases in which the voltage was mapped using the radial minielectrodes together with the details provided by the use of EAM.

The disparity of procedure success in patients presenting for ablation in atrial flutter has previously been observed by Subbiah et al.^10^ Here, the authors demonstrated that sinus rhythm at the start of ablation was independently associated with short ablation times [odds ratio: 8.03; p = 0.005]. While this initially appears counterintuitive, as one would expect the bundles employed during atrial flutter to be readily identified during the arrhythmia, there are a number of confounding variables: (1) the direction of propagation during coronary sinus pacing, sinus rhythm, and atrial flutter are very different and thus affect the voltage profile of the isthmus (as well as the voltage values themselves). Furthermore, the initial target in CTI ablation is clockwise conduction, as manifested during pacing from the coronary sinus. This conduction vector is in the opposite direction to that seen during typical, counterclockwise atrial flutter and may employ different muscle bundles or concealed conduction. If the voltage is altered and this is employed to target muscle bundles, then the “less-forgiving” MiFi™ catheter (Boston Scientific, Natick, MA, USA), already centered away from the putative bundle, would do particularly badly given the location mismatch explained. Additionally, (2) catheter stability/contact during the rapid atrial contractions of flutter may be less than that during sinus rhythm. The mean temperature and variability in power between lesions might also be an indicator of this.

The intent of the minielectrodes is to provide a measure of contact and accuracy, and there are studies supporting this.^4^ Moreover, in a traditionally perpendicular orientation, the 8-mm MiFi™ catheter (Boston Scientific, Natick, MA, USA) performed well as found by ourselves in the ablation of an atriofascicular pathway.^11^ These data presented illustrate the importance of the location of the recording electrodes and the catheter’s orientation. A similar distal electrode would reduce the mismatch and one might anticipate a more optimal result; however, this requires prospective study.

## Summary

While IntellaTip MiFi™ technology (Boston Scientific, Natick, MA, USA) is highly effective at detecting the small voltage signals in redo atrial flutter ablation and atriofascicular pathway ablation, the 8-mm nonirrigated-tip catheter does not enhance voltage-directed CTI ablation. The same may not be true for the 4-mm irrigated platform. Further studies on this will be required.

## Figures and Tables

**Table 1:** Demographics and Procedure Details of the Two Groups of Patients

	Group A: Conventional Bipole (n = 13)	Group B: Minielectrodes (n = 17)	p-value
Age	66 years ± 2 years	64 years ± 3 years	0.24
Diabetes mellitus	8%	22%	0.38
Hypertension	46%	29%	0.49
LV ejection fraction	44.5% ± 2.5%	47.6% ± 3.2%	0.32
RF time	576 s ± 76 s	638 s ± 108 s	0.34
Number of RF applications	10 ± 1.4	13 ± 2.2	0.13
Mean power	44.8 W ± 1.9 W	38.7 W ± 2.0 W	0.04
Mean temperature	52.6°C ± 0.9°C	53.7°C ± 1.0°C	0.23
Fluoroscopy time	15.8 min ± 2.0 min	21.5 min ± 3.0 min	0.06
Procedure time	82 min ± 4.8 min	100.6 min ± 11.8 min	0.10

n: number; LV: left ventricular; RF: radiofrequency.

**Table 2:** A Comparison of Ablation Parameters in Patients Presenting for Ablation in Atrial Flutter

	Group A: Conventional Bipole (n = 6)	Group B: Minielectrodes (n = 8)	p-value
RF time	449 s ± 120 s	719 s ± 194 s	0.15
Number of RF applications	7.8 ± 1.1	15.4 ± 4.0	0.07
Mean power	46.0 W ± 2.9 W	37.1 W ± 2.3 W	0.03
Mean temperature	51.8°C ± 0.6°C	55.2°C ± 1.1°C	0.02
Fluoroscopy time	13.4 min ± 2.7 min	26.3 min ± 4.8 min	0.03
Procedure time	73 min ± 6.9 min	115.7 mins ± 17.7 mins	0.03

n: number; RF: radiofrequency.

**Table 3:** A Comparison of Ablation Parameters in Patients Presenting for Ablation in Sinus Rhythm

	Group A: Conventional Bipole (n = 7)	Group B: Minielectrodes (n = 9)	p-value
RF time	684 s ± 104 s	573 s ± 149 s	0.29
Number of RF applications	11.8 ± 2.4	11.3 ± 2.8	0.44
Mean power	43.9 W ± 3.3 W	39.0 W ± 4.3 W	0.21
Mean temperature	53.4°C ± 1.7°C	53.9°C ± 1.4°C	0.41
Fluoroscopy time	17.9 min ± 3.3 min	19.2 min ± 4.7 min	0.41
Procedure time	90.3 min ± 6.6 min	92.5 min ± 20.7 min	0.46

n: number; RF: radiofrequency.
